# Patterns of Conservation and Loss of Hox Genes in Xenacoelomorph Lineage Since Divergence From Last Common Bilaterian Ancestor

**DOI:** 10.1093/gbe/evag094

**Published:** 2026-04-10

**Authors:** Ariane Buckenmeyer, Samuel Abalde, Joseph F Ryan, Ulf Jondelius

**Affiliations:** Department of Zoology, Swedish Museum of Natural History, Stockholm, Sweden; Department of Gene Technology, Science for Life Laboratory, KTH – Royal Institute of Technology, Solna, Sweden; Department of Zoology, Swedish Museum of Natural History, Stockholm, Sweden; Atmosphere and Ocean Research Institute, The University of Tokyo, Kashiwa, Japan; Whitney Laboratory for Marine Bioscience, University of Florida, St. Augustine, FL, USA; Department of Biology, University of Florida, Gainesville, FL, USA; Department of Zoology, Swedish Museum of Natural History, Stockholm, Sweden

**Keywords:** Xenacoelomorpha, early Bilateria, Hox, homeobox, motif enrichment

## Abstract

The explosion of body forms found in bilaterians is thought to be tied to the diversification of Hox transcription factors, which play a critical role in development along the anterior-posterior axis for most bilaterians. However, the evolutionary history of Hox genes in Bilateria's early branches remains unclear. Xenacoelomorpha, a clade of marine worms including *Xenoturbella* and the acoelomorphs Acoela and Nemertodermatida, have a simple Hox complement and are a particular group of interest. Past surveys of Hox in Xenacoelomorpha have been taxonomically limited. To address this, we analyze the homeodomains and surrounding amino acid motifs coded by Hox, ParaHox, and extended Hox genes across 40 xenacoelomorph transcriptomes and four genomes, along with representatives of other major bilaterian groups and the anthozoan *Nematostella vectensis*. We show that several motif annotations previously proposed to be synapomorphies of Acoela are ubiquitous across both Xenacoelomorpha and the rest of Bilateria and found new Hox content specific to Acoela and *Xenoturbella*. Our results reveal homology between xenacoelomorph antHox and other bilaterian Hox1 genes, though the relationships of centHox and postHox genes remain unresolved either due to rapid sequence evolution or extensive birth-death processes. Our analyses suggest that diversification of bilaterian Hox involved both extensive retention and loss of ancestral content. Hox variability between xenacoelomorphs and other bilaterians reflects selective retention of ancestral Hox content rather than affinity to either deuterostomes or protostomes and does not remove support from a monophyletic Xenacoelomorpha sister group to all other Bilateria.

Significance Hox genes are central to antero-posterior patterning across Bilateria, yet their diversity in Xenacoelomorpha—which features a simpler Hox complement compared to most other bilaterians—remains poorly characterized. Using the broadest taxonomic coverage of xenacoelomorphs to date, we were able to establish baseline complements for acoelomorph and xenoturbellid Hox, ParaHox, and extended Hox genes, which were compared to existing references in other bilaterians and *Nematostella vectensis*. In addition to the 60 aa homeodomain, we annotated motif content across Xenacoelomorpha and our reference set to assess the relationship between xenacoelomorph and other bilaterian Hox genes. We recovered strong support for xenacoelomorph antHox as homologous to bilaterian Hox1, but poor support for central and posterior Hox genes. The heavy conservation of Hox homeodomain and motif content shared between both groups suggests substantial retention of ancestral bilaterian features, while lineage-specific variation indicates that both gene divergence and loss shaped extant xenacoelomorph HOX-L content.

## Introduction

The diversification of bilaterian body plans is assumed to be closely tied to the evolutionary history of Hox genes (Lewis 1978; Kappen and Ruddle 1993), a group of transcription factors that emerged prior to the divergence (Kourakis and Martindale 2000; Ferrier and Holland 2001; Kamm et al. 2006; Chourrout et al. 2006; Ryan et al. 2007) of Bilateria from its sister group Cnidaria (Kim et al. 1999; Hejnol et al. 2009; Technau and Steele 2011). Hox genes belong to the Antennapedia (ANTP) class of the homeobox superfamily (Holland et al. 2007), which is characterized by the presence of a static 60 aa DNA-binding homeodomain (Scott et al. 1989) which has remained remarkably conserved since the diversification of bilaterians during the Cambrian (Valentine 1994; Holland 2015). These genes are critical to determining body plan specification along the primary body axis (Lewis 1978; McGinnis and Krumlauf 1992) and form a crucial part of the broader developmental toolkit found in most animals (Carrasco et al. 1984; Gehring 1985; Murtha et al. 1991; Dessain et al. 1992; Carroll 1995; Ferrier and Holland 2001; Lemons and McGinnis 2006). Two clusters of the ANTP class homeobox genes, the Hox (Holland 2013) and ParaHox (Brooke et al. 1998) subclasses, hold particularly important roles for animal development and the evolution of structural novelties (Pick and Heffer 2012; Ferrier 2016). Hox genes are highly diversified and primarily regulate development and regionalization on the anterior-posterior (AP) axis (Garcia-Fernàndez 2005), while the closely related ParaHox (Brooke et al. 1998) and “extended Hox” (Hox-linked or Hox-like) genes (Minguillón and Garcia-Fernàndez 2003) are more involved in the regional specification of developing structure and tissue (Gehring et al. 1994; Gaunt et al. 1988; Urbach and Technau 2003; Zhong et al. 2020). Hox and ParaHox genes are often arranged in genomic clusters whose organization governs spatial and temporal patterning (Ferrier and Minguillón 2003; Garcia-Fernàndez 2005), but the degree of clustering—ranging from intact to disorganized to fully dispersed (Duboule 2007)—can vary markedly across animal groups (Ferrier and Holland 2002; Hui et al. 2008). While the different bilaterian lineages exhibit substantial change in Hox gene content and genomic presentation since their initial origin (Brooke et al. 1998; Paps and Holland 2018; Guijarro-Clarke et al. 2020), the core sequence of the homeodomain has remained remarkably conserved (McGinnis et al. 1984; Murtha et al. 1991), making the evolutionary history of Hox genes difficult to reconstruct (Garcia-Fernàndez 2005; Ferrier 2010). In addition, the Hox homeodomain, similar to other transcription factor groups (Levine and Hoey 1988), are frequently accompanied by short peptide motifs around the homeodomain N- and C-terminals (Bürglin 1997) that contain characteristic patterns that facilitate sequence selection and DNA-binding specificity of Hox proteins (Chauvet et al. 2000; Pick and Heffer 2012). These motif patterns are often highly diverse and lineage-specific and may provide additional phylogenetic information on the evolutionary relationships of the different bilaterian Hox genes (Bayascas et al. 1998; Fröbius and Funch 2017; Szabó and Ferrier 2018).

The body plan of Xenacoelomorpha presents a stark contrast to the wide array of highly specialized and differentiated traits characteristic of most Bilateria (Jondelius et al. 2019). Current analyses generally support that Xenacoelomorpha consists of Acoelomorpha (with Acoela positioned as sister clade to Nemertodermatida) and *Xenoturbella* (Hejnol et al. 2009; Rouse et al. 2016; Cannon et al. 2016; Laumer et al. 2019; Álvarez-Presas et al. 2024; Liu et al. 2024), but an alternative scenario has been proposed questioning the monophyly of Acoelomorpha and instead grouping Acoela with *Xenoturbella* (Redmond 2024). Xenacoelomorpha's position within Bilateria is also contested, with alternate analyses recovering xenacoelomorphs in different positions across the metazoan tree of life (Haszprunar 1996; Jondelius et al. 2002; Ruiz-Trillo et al. 2002; Ruiz-Trillo et al. 2004; Wallberg et al. 2007; Dunn et al. 2008; Philippe et al. 2011; Haszprunar 2016; Philippe et al. 2019; Marlétaz et al. 2019). The last two decades have seen two major hypotheses dominating the discussion regarding their relationship to other bilaterian animals. Under the Nephrozoa hypothesis, Xenacoelomorpha are the sister group to all other bilaterians (the Nephrozoa hypothesis) (Cannon et al. 2016; Álvarez-Presas et al. 2024), and their early-diverging status explains the absence of many bilaterian characteristics such as excretory organs, body cavity, and a through gut. Conversely, the Xenambulacraria hypothesis presents Xenacoelomorpha as the sister group to Ambulacraria (Philippe et al. 2011; Philippe et al. 2019) with Chordata sister to Lophotrochozoa + Ecdysozoa rendering deuterostomes paraphyletic (Kapli et al. 2021) and implying absence of many bilaterian features as the result of multiple secondary losses. It is possible that the Hox gene complements in contemporary xenacoelomorphs are informative of their disputed evolutionary trajectory (Baguñà and Riutort 2004; Sikes and Bely 2010; Thomas-Chollier and Martinez 2016; Brauchle et al. 2018; Wanninger 2024).

There is limited data on Hox complements in Xenacoelomorpha. Only three out of 23 Acoela families have been surveyed, along with a single species of Nemertodermatida, *Nemertoderma westbladi*, and *Xenoturbella bocki*. It has therefore been difficult to establish a hypothesis on the condition of the ancestral acoelomorph Hox complement and subsequent evolution. Available data indicates similarity to the canonical HOX-L (“HOX-Like,” ex. Hox, ParaHox, and extended Hox genes) complement of the other bilaterians (Holland et al. 2007), with single Hox genes belonging to an anterior group (antHox), a central group (centHox), a posterior group (postHox), a ParaHox group (Cdx & Gsx) (Cook et al. 2004; Jiménez-Guri et al. 2006; Hejnol and Martindale 2009) (Table 1), and typically single genes for the “extended Hox” (Evx, Gbx, Mox, Mnx, and Ro) (Brauchle et al. 2018). A common feature of Hox genes is their organization in the genome as collinear genomic clusters (Duboule 2007). While this structure is maintained in *Xenoturbella* (Schiffer et al. 2024), acoel Hox genes are atomized and otherwise scattered across the genome (Hejnol and Martindale 2009; Moreno et al. 2009; Moreno et al. 2011; Gaunt 2018). A loss of Hox collinearity has also been proposed for Nemertodermatida, but this is yet to be tested (Moreno et al. 2011).

**Table 1:** Previous Hox, ParaHox, and extended Hox (“HOX-L”) studies involving xenacoelomorphs

Organism	antHox	centHox	postHox	Cdx	Gsx	Xlox	Evx	Mox	Mnx	Gbx	Ro
*Aphanostoma pulchra*	1^6,8,9^	1^6,8,9^	1^6,8,9^	1^8^	1^8^	…	1^8,9^	1^8^	1^8^	…	1^8^
*Convolutriloba longifissura*	1^6^	1^6^	1^6^	1^5,6^	…	…	1^5^	…	…	…	…
*Hofstenia miamia*	1^8,9^	1^8,9^	1^8,9^	1^8^	1^8^	…	1^8,9^	…	1^8^	…	2^8^
*Paratomella rubra*	…	1^1^	2^3^	1^1,3^	…	…	…	…	…	…	…
*Praesagittifera naikaiensis*	1^9^	1^9^	1^9^	…	…	…	…	…	…	…	…
*Symsagittifera roscoffensis*	1^1,2,6,7,8,9^	1^1,2,3,6,7,8,9^	1^2,3,6,7,8^	1^2,3,6,8^	1^8^	…	1^8^	…	1^8^	…	1^8^
*Nemertoderma westbladi*	1^8,9^	2^3^	1^3,8,9^ 2^8,9^	1^3,8^	…	1^3^	2^8^	1^8^	1^8^	1^8^	1^8^
*Xenoturbella bocki*	1^4,8,9,10^	2^10^ 3^4,8,9^	1^4,8,9,10^	1^8,10^	1^8^ 2^10^	…	1^8,10^	1^8^	1^10^	1^8^	…

Number of Hox, ParaHox, and extended Hox genes annotated in previous surveys. Here, we use the labels anterior Hox (antHox), central Hox (centHox), and posterior Hox (postHox) whereas ParaHox (Cdx, Gsx, and Xlox) and extended Hox (Evx, Meox/Mox, Mnx, Gbx) and Ro genes are named according to their corresponding bilaterian gene family. Studies numbered sequentially. (1) Saló et al. (2001), (2) Cook et al. (2004), (3) Jiménez-Guri et al. (2006), (4) Fritzsch et al. (2008), (5) Hejnol and Martindale 2008, (6) Hejnol and Martindale 2009, (7) Moreno et al. (2009), (8) Brauchle et al. (2018), (9) Ueki et al. (2019), and (10) Schiffer et al. (2024). *Symsagittifera roscoffensis* in Saló et al. (2001) was then referred to as *Convoluta roscoffensis*. *Aphanostoma pulchra* was previously referred to as *Isodiametra pulchra.*

Conservation of the Hox homeodomain has made it difficult assessing the orthology of xenacoelomorph and nephrozoan Hox genes. A Hox gene tree may help address the relationship between the xenacoelomorph set of Hox genes and those of other bilaterians, while Hox motifs may allow more lineage-specific analyses, and more specifically, the detection of duplication events and shared ancestral content. However, few studies have reviewed the composition of acoel (Cook et al. 2004; Ogishima and Tanaka 2007; Moreno et al. 2009) or xenacoelomorph Hox motifs (Ueki et al. 2019), and none have examined motifs in xenacoelomorph ParaHox and extended Hox.

Improved taxonomic sampling of xenacoelomorph Hox data will help facilitate reconstruction of the ancestral xenacoelomorph Hox complement. Here, we applied new transcriptomic and genomic data to survey Hox across a broader phylogenetic diversity of Xenacoelomorpha and reassess the accuracy of previous xenacoelomorph Hox, ParaHox, extended Hox gene, and motif annotations. Our approach used a Hox phylogeny built on 60 amino acid homeodomains of xenacoelomorphs, nephrozoans, and an anthozoan to examine homology between the different groups. We additionally surveyed the presence of potentially informative amino acid motifs flanking the 20 amino acids next to the Hox homeodomain to gauge the reliability of motif phylogenetic signal for resolving Xenacoelomorpha's position.

## Results

### The Xenacoelomorph Hox Complex

**Fig. 1.**
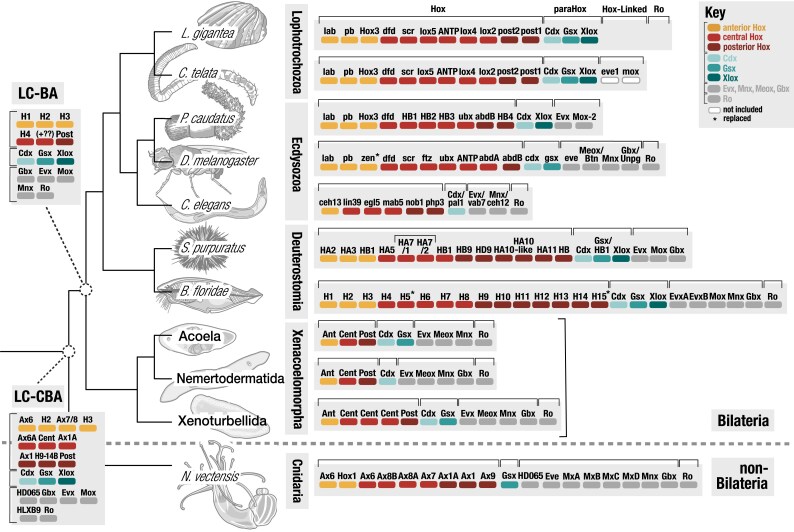
Characterization of HOX-L diversity of Xenacoelomorpha as compared to Anthozoa and Nephrozoa (under the “Nephrozoa” hypothesis). Hox (antHox, centHox, postHox), ParaHox (Cdx, Gsx, Xlox/Pdx), and extended Hox (Gbx, Evx, Mox, Mnx, Ro) gene diversity across major metazoan lineages: Cnidaria (the anthozoan *N. vectensis*), Xenacoelomorpha (incl. study species from Acoela, Nemertodermatida, *Xenoturbella*), Deuterostomia (the chordate *B. floridae* and echinoderm *S. purpuratus*), and Protostomia, composed of Ecdysozoa (the priapulid *Priapulida caudatus*, arthropod *D. melanogaster*, and nematode *C. elegans*) and Spiralia (the mollusk *L. gigantea* and annelid *C. teleta*). Blank spaces imply absence of a gene annotation at the time of this survey. Organization of Hox, ParaHox, and extended Hox genes implies only presence, not clustering or order.

Our results support an acoelomorph Hox complement that typically consists of at least one anterior, central, and posterior genes (Fig. 1) and a xenoturbellid Hox complement consisting of an anterior, three central (centHox1, centHox2, and centHox3) (Fig. 1), and one posterior gene. The coverage and retention of ParaHox and extended Hox genes displayed greater variability across xenacoelomorphs (Supplementary material), although the baseline appears to be a ParaHox complement including a Cdx, Gsx, and an absence of a Xlox/Pdx homolog and an extended Hox complement including an Evx, Meox, Mnx, Ro, and Gbx. We note some exceptions to this rule and observed an absence of a nemertodermatid Gsx gene and an acoel Gbx gene. We also observed the following instances in acoelomorphs where we observed more than a single gene per HOX-L family: (i) *Paratomella rubra* (postHox1 and postHox2 and Cdx1 and Cdx2), (ii) *Hofstenia miamia* (Ro1 and Ro2), (iii) nemertodermatid *N. westbladi (*postHox1 and postHox2 and Evx1 and Evx2) (Fig. 1), and (iv) two *Xenoturbella* Gsx genes (Gsx1 and Gsx2).

### Tree Topology

**Fig. 2.**
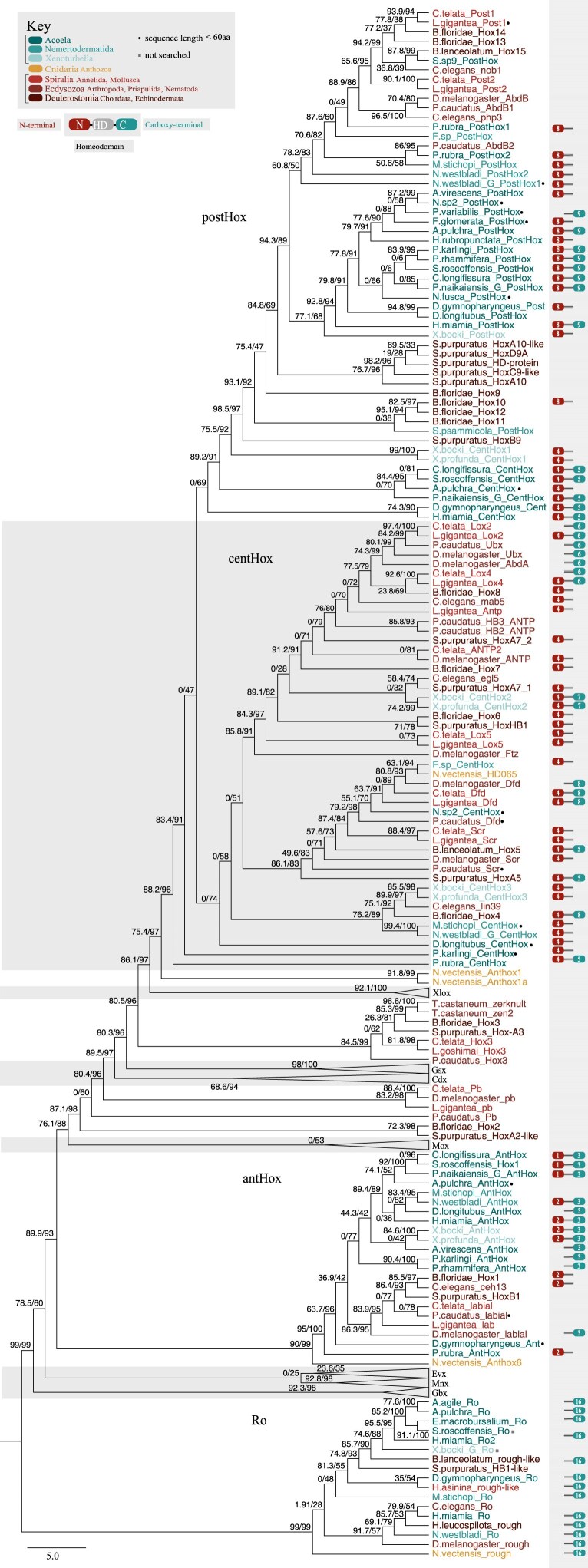
Maximum likelihood (ML) phylogenetic tree of Hox genes. ML phylogenetic tree of Hox genes (anterior, central, and posterior) under the LG + G4 model. Labels at nodes show IQTree UF bootstrap frequencies. Phylogenetic tree was built on the 60 aa homeodomain of HOX-L genes and rooted with Ro. Different xenacoelomorph taxa are color-coded in shades of blue (acoels: dark blue, nemertodermatids: medium blue, xenoturbellids: light blue), the anthozoan *N. vectensis* in yellow, and nephrozoan taxa in shades of red (deuterostomes: dark red, ecdysozoans: medium red, spiralians, light red). Motif colors correspond with the N-terminus (red) and C-terminus (blue) bordering the homeodomain. N- and C- terminal motifs are labeled numerically according to Fig. 4. Full HOX-L tree available in the Supplementary material.

Our phylogenetic analyses included representatives of roughly 60% of xenacoelomorph families (Supplementary material). Despite the limited phylogenetic signal of the 60 aa homeodomain, we identified several patterns among xenacoelomorph, nephrozoan, and anthozoan Hox genes. We found strong support (BS = 95%) for a homologous relationship between the xenacoelomorph anterior Hox and nephrozoan Hox1 (Fig. 2). The relationships between the xenacoelomorph and nephrozoan central and posterior Hox genes were less resolved. The central Hox genes were not recovered as monophyletic despite high confidence in the gene annotations. The majority of acoel centHox genes clustered with the *Xenoturbella* centHox1, while others grouped with *Xenoturbella* centHox3 and nephrozoan Hox4 and Hox5 (Fig. 2). The *Xenoturbella* centHox2 genes were recovered within the nephrozoan central Hox clade with Hox6-8 and Hox7. A group of acoel and *Xenoturbella* postHox genes, along with a group of acoelomorph and protostome postHox, formed a clade (BS = 94.3%) together, while deuterostome posterior Hox genes were recovered separately (Fig. 2). In both cases, most centHox and postHox genes recovered outside of the main Xenacoelomorpha clade correspond to partial sequences, which may have limited the already low phylogenetic signal and complicated homology inference for these genes.

### Shared Motif Content Between Xenacoelomorphs and Nephrozoans

We searched for potentially diagnostic motif content in the 20-amino acid N-terminal and C-terminal regions flanking the Hox, ParaHox, and extended Hox homeodomains using the MEME motif recognition program. We detected a high proportion of shared motif content between xenacoelomorphs, nephrozoans, and two shared motifs with *N. vectensi*s and made additional observations of possible lineage-specific patterns within Xenacoelomorpha and Nephrozoa. Our analysis excluded motifs located beyond the 20-amino acid cutoff (with some additional patterns of interest detailed in the Supplementary material), and we add that the absence of motifs here should be interpreted cautiously as they may be identified in future work.

We observed a particularly high level of motif content shared between xenacoelomorphs and nephrozoans. Three motifs were ubiquitous across the major branches of both clades: the central Hox WM hexapeptide/PBX motif and the Cdx TGKTRT and the Ro KAQLDQQ motifs (Fig. 4). The WM motif can be found in the 20-amino acid region preceding the homeodomain in most bilaterian central Hox genes, as well as in *N. vectensis* Anthox6A, Anthox7, and Anthox8B (Figs. 2 and 4; Supplementary material). The two amino acids preceding the WM motif may represent a lineage-specific pattern: the FA(WM) variant, specific to most acoels, and the more common YP(WM) found in other xenacoelomorphs (*P. rubra*, *D. gymnopharyngeus*, *M. stichopi*, *Flagellophora* sp., and *X. profunda*), protostomes, deuterostomes, and *N*. *vectensis*. The Cdx motif TGKTRT was conserved across four acoels, *M. stichopi*, *X. profunda*, and the nephrozoans *C. teleta*, *L. gigantea*, *Drosophila melanogaster*, *P. caudatus*, and *S. purpuratus* (Fig. 4). We annotated the Ro motif KAQLDQQ across all three branches of xenacoelomorphs and nephrozoans and *N. vectensis*. There were several other less ubiquitous residues notable for their shared nephrozoan-xenacoelomorph motif content. The antHox motifs KRSQP and KEGKL were broadly represented amongst acoels, *N. westbladi*, and *Xenoturbella* and additionally in the Hox1 genes of the nephrozoans *C. elegans* and *B. floridae*, and *D. melanogaster*, respectively (Figs. 2 and 4). The centHox NLKSMSQ motif was similarly well-conserved between acoels and the Hox5 and HoxA5 genes of the deuterostomes *B. lanceolatum* and *S. purpuratus* (Figs. 2 and 4). The postHox WL/WM motif was shared across all major xenacoelomorph branches and the Hox10 gene of the deuterostome *B. floridae*. The Cdx motif RK is shared between all three branches of xenacoelomorphs and the protostomes *C. elegans* and *C. teleta*, while the Evx motif WPYLDPYYYAY is present in all three branches of xenacoelomorphs, the protostome *P. caudatus*, and the deuterostome *B. floridae*. Interestingly, we annotated no motifs exclusive to all three branches of Xenacoelomorpha without being present in at least one nephrozoan.

### Lineage-Specific “Diagnostic” Hox Motifs Within Xenacoelomorpha

Despite the amount of shared Hox content, we also noted lineage specificity possibly informative of the distinct evolutionary history of those genes. We observed that our “nephrozoan-specific” motifs were exclusive to centHox and Xlox genes, while the different xenacoelomorph clade-specific motifs were dispersed amongst the anterior, central, and posterior Hox and Meox genes (Fig. 4). Several of these motifs appear conserved to specific clades within Acoela, for example, the antHox GCHLGPGVQ motif (Convolutidae) and the Meox KRRGVRTASIGEGGMNECEE motif (Dakuidae), and a collection of several conserved patterns within the posterior Hox motifs (Fig. 4). We also observed this in *Xenoturbella*, identifying a unique motif signature associated with the *Xenoturbella* centHox2 (KIGNKRSVDYAESGSTSPPS). The high proportion of lineage-specific Hox content found in Acoela and *Xenoturbella* was not reflected in our current analyses of Nemertodermatida (Fig. 4).

## Discussion

Although the evolution of Hox genes has been widely studied across extant Bilateria, the coverage of available homeodomain content in Xenacoelomorpha has been sparse (Moreno et al. 2011; Brauchle et al. 2018; Ueki et al. 2019). This survey expands upon previous studies with additional transcriptomic and genomic data, representing the broadest taxon coverage of xenacoelomorph Hox, including ParaHox and other associated Hox genes to date. The updated evidence of shared HOX-L homeodomain and motif content between xenacoelomorph and bilaterian animals allows for a reevaluation of multiple points on previous Hox annotations, homologies, and the potential phylogenetic signal in Hox motif content. We only interpreted absences of specific genes as lineage-specific losses when corroborated by genome data. For example, the absence of antHox in *Flagellophora* sp. and *Sterreria psammicola* is more likely attributed to low transcriptome completeness (Supplementary material) rather than independent losses in Ascopariidae and Nemertodermatidae. That said, our results support the inclusion of transcriptome data as a cost-effective and reliable method for broadening taxonomic coverage of Hox data in an under-sampled group.

### Variation in Hox, ParaHox, and Extended Hox Genes Across Xenacoelomorpha

Our results support an acoelomorph Hox complement consisting of a single anterior, central, and posterior gene as described in the previous surveys on *H. miamia* (Brauchle et al. 2018), *Aphanostoma pulchra* (Hejnol and Martindale 2009; Brauchle et al. 2018), *Convolutriloba longifissura* (Hejnol and Martindale 2009), *Symsagittifera roscoffensis* (Cook et al. 2004; Moreno et al. 2009; Hejnol and Martindale 2009; Brauchle et al. 2018), and *Praesagittifera naikaiensis* (Arimoto et al. 2019). Our results also corroborated dual genes for *N. westbladi* postHox and Evx, *H. miamia* Ro, and *P. rubra* postHox previously reported by Brauchle et al. (2018) and Jiménez-Guri et al. (2006), alongside a new annotation of *N. westbladi* centHox and a second *P. rubra* Cdx, reinforcing the need of extended taxon sampling for describing Hox diversity. Like acoelomorphs, *Xenoturbella* also has a single anterior and posterior gene, while the triad of central Hox genes we annotated in *X. profunda* is consistent with the existing *X. bocki* annotations (Brauchle et al. 2018; Schiffer et al. 2024) and appears to be a characteristic of *Xenoturbella*. The expanded taxon coverage in our survey corroborates the widespread loss of an Xlox/Pdx ortholog in Xenacoelomorpha (Brauchle et al. 2018; Schiffer et al. 2024). The presence of Gsx in acoels and in *Xenoturbella*, while it is absent in the *N. westbladi* genome, suggests loss of this gene in Nemertodermatida, while the absence of an acoel Gbx is consistent with a loss within Acoela (Brauchle et al. 2018).

Although our results are mostly consistent with the recent work done on *N. westbladi* (Brauchle et al. 2018) and *X. bocki* (Schiffer et al. 2024), the availability of new data also helped update annotations to the *N. westbladi* centHox and Evx and *X. bocki* Gsx genes. The initial two *N. westbladi* 26/28 aa centHox annotations by Jiménez-Guri et al. (2006) were later attributed to mollusk contamination (Thomas-Collier and Martinez 2016; Thomas-Collier, unpublished), and subsequent studies (Brauchle et al. 2018; Ueki et al. 2019) corroborated the absence of centHox. This is consistent with our observation of both *N. westbladi* centHox annotations being identical to Hox5/Scr and Hox7/Lox4 of the mollusk *Lottia gigantea* in our reference dataset. However, our analyses of the *N. westbladi* genome (Abalde et al. 2023) recovered a new 45 aa centHox sequence distinct from the annotations presented in Jiménez-Guri et al. (2006) and reestablishes support for a centHox gene in Nemertodermatida. Jiménez-Guri et al. (2006) annotated Xlox in *N. westbladi*, with high sequence similarity to Xlox in the mollusk *L. gigantea*, which re-raises the issue of a mollusk contamination as no other Xlox was annotated in any xenacoelomorphs in subsequent studies (Moreno et al. 2011; Brauchle et al. 2018; Schiffer et al. 2024). Considering the absence of a Xlox annotation in the genome data, it is possible that the absence of Xlox, which is central to mid-gut patterning in other bilaterians (Brooke et al. 1998; Hui et al. 2009), is linked to the gut morphology in Xenacoelomorpha where all species lack a through gut and Acoela do not even have a gut cavity (Hejnol and Martindale 2008).

Each *N. westbladi* dataset also revealed unique copies of the Evx genes: Evx1 in the genome and Evx2 in the transcriptome, both consistent with the dual *N. westbladi* Evx annotations presented in Brauchle et al. (2018). Likewise, we also annotated a second *Xenoturbella* Gsx gene referenced in Schiffer et al. (2024), which was previously absent in transcriptomic work (Brauchle et al. 2018).

This extended dataset can also address the nature of Hox organization in the genome. Hox genes (along with ParaHox and extended Hox genes) were annotated in the *N. westbladi* genome (Abalde et al. 2023) in separate contigs (Supplementary material) and flanked by other genes. This confirms the absence of Hox collinearity in Nemertodermatida, a condition previously reported in Acoela (Hejnol and Martindale 2009; Moreno et al. 2009; Moreno et al. 2011; Gaunt 2018).

### Implications of Duplicate Hox Genes in Acoelomorpha and *Xenoturbella*

The duplicate Hox genes reported here, and in *H. miamia* (Brauchle et al. 2018), *P. rubra* (Cook et al. 2004; Moreno et al. 2011), *N. westbladi* (Brauchle et al. 2018), and *Xenoturbella* (Brauchle et al. 2018; Schiffer et al. 2024) are points of interest on the Hox content of the ancestral xenacoelomorph. There are several ways to explain these additional copies of Hox genes, which could reflect a whole genome duplication (WGD) event, a species-specific gene duplication, or the retention of multiple ancestral genes. Although Hox genes can be effective indicators for WGD (Holland 2013; Bürglin and Affolter 2016), the conserved homeodomain and motif content of postHox and Evx paralogs argue against it in *N. westbladi* (Abalde et al. 2023), and while the large repetitive genomes found in acoels could be compatible with past WGD, available genomes (Arimoto et al. 2019; Gehrke et al. 2019; Martinez et al. 2023) provide no evidence for it.

The duplicate postHox and Cdx genes in *Paratomella* and the duplicate Ro genes in *Hofstenia*, with all of them closely positioned to one another and sharing at least one motif with their pair (Supplementary material), are most likely lineage-specific duplications of a single ancestral gene. Likewise, the close placement of the duplicate *X. bocki* Gsx genes (Schiffer et al. 2024) implies that they are also the result of independent gene duplications. The triad of the central Hox genes, however, might have arisen from a single ancestral gene, later duplicated in *Xenoturbella*, or inherited from the Last Common Bilaterian Ancestor (LC-BA) and subsequently lost in Acoelomorpha. The disparate placements of the *Xenoturbella* centHox genes in relation to one another support their ancestral condition, especially in light of a motif and pattern unique to *Xenoturbella* centHox2 and centHox3, respectively (Supplementary material), but this scenario should be revisited with more in-depth analysis of *X. bocki*'s genome data (Schiffer et al. 2024).

### Shared Motifs Suggest Deep Conservation of Hox Content Throughout Bilateria

The conservation of Hox motifs is often highlighted as a potential source of phylogenetic signal (Bayascas et al. 1998; de Rosa et al. 1999; Galant and Carroll 2002; Balavoine et al. 2002; Moreno 2010; Fröbius and Funch 2017). The use of Hox signatures for phylogenetic placement of acoels was first mentioned in Adoutte et al. (2000), referencing unpublished work identifying lophotrochozoan Hox signatures in the acoel *Childia*, but this was never revisited. It is important to highlight that the use of “diagnostic” HOX-L motifs as reliable phylogenetic markers is contingent on taxonomic coverage. A case in point is the antHox KEGKL (Ueki et al. 2019) and centHox NLKMSQ (Deutsch 2008; Ueki et al. 2019) motifs. These were initially considered to be diagnostic for xenacoelomorph and acoel Hox genes but are here reported also in the protostome *D. melanogaster* Hox1/Lab gene (Singh et al. 2020), and the deuterostome *B. lanceolatum* Hox5 and *S. purpuratus* HoxA5 genes, respectively. The overlap of several of these acoel motifs with nephrozoan content was also previously observed in Hejnol and Martindale (2009) under a similar comparison of acoel, bilaterian, and cnidarian PBX, N-terminal, and C-terminal Hox sequences. We annotated several putative lineage-specific motifs (four to Acoela and two to *Xenoturbella*), including the antHox GCHLGPGVQ in Convolutidae. If this motif is truly specific to this family, it would be consistent with the rapid evolution rates and numerous character novelties associated with the convolutid acoels (Jondelius and Jondelius 2019; Abalde and Jondelius 2025). Nonetheless, it remains open that this and many of the motifs we consider here to be “lineage-specific” are identified in other bilaterians in the future. While this instability makes Hox motif content unsuitable for assessing Xenacoelomorpha's phylogenetic placement, they may be informative on the Hox homology of the ancestral bilaterian. The motif content shared between Xenacoelomorpha and Nephrozoa, such as the ubiquitous YPWM hexapeptide (Deutsch 2008; Pick and Heffer 2012; Hubert and Wellik 2023) visible here in central Hox, the Cdx TGKTRT, or the Ro KAQLDQQ (Fig. 4), suggests their presence in the ancestral Hox of the LC-BA and their retention throughout the radiation of Bilateria. Likewise, the origins of lineage-specific motifs such as the tentatively dakuid-specific Meox KRRGVRTASIGEGGMNECEE may represent new innovations in that lineage, in this case was gained after the radiation of the Last Common Acoel Ancestor (LC-AcA). Our MEME motif analyses focused strictly on the 20 amino-acid region flanking the Hox homeodomain, as many paralog and cofactor-specific interaction sites and features (Rorick and Wagner 2010; De Kumar and Darland 2021), are often found nearest to the homeodomain (Fröbius and Funch 2017; Szabó and Ferrier 2018). While the N- and C- terminal tails beyond that are far more variable in length and sequence identity, it should not be excluded that additional motifs may be located beyond the window of this study.

### Xenacoelomorph antHox Gene Is Homologous to Nephrozoan Hox1

One of the primary aims of this survey was to establish homology between xenacoelomorph and nephrozoan Hox genes. Our results support homology of the single xenacoelomorph antHox gene and the nephrozoan Hox1 gene, which is additionally supported by the presence of the antHox motifs KRSQPNAVSK in *B. floridae* Hox1 and *C. elegans* ceh13/Hox1 genes and the KEGKL/CTM-motif annotated in the Hox1/lab gene of *D. melanogaster* in Singh et al. (2020). Our results also support the absence of a homolog to the nephrozoan Hox2 and Hox3 genes (Moreno and Martinez 2010; Brauchle et al. 2018; Wanninger 2024), meaning that they were possibly lost in the ancestor of Xenacoelomorpha, as suggested by Schiffer et al. (2024), or appeared after the radiation of Bilateria (Brooke et al. 1998). This is further supported by the close relationship of *N. vectensis* Anthox6 and bilaterian Hox1, which is consistent with previous work (Steinworth et al. 2023; Ryan et al. 2007), but links between other *N. vectensis* Hox and nephrozoan Hox2 and Hox3 remain unclear (Steinworth et al. 2023).

### Reconstruction of Ancestral Hox State of Xenacoelomorpha

**Fig. 3.**
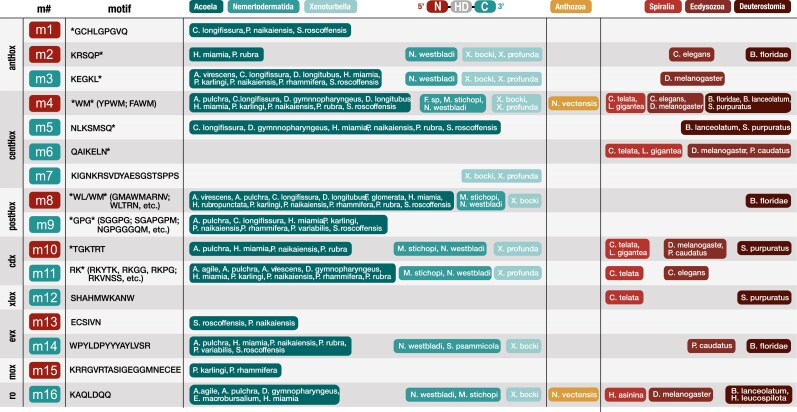
Hox, ParaHox, and extended Hox motif enrichment across Xenacoelomorpha, Nephrozoa, and *N. vectensis.* Motifs identified by MEME from the 20 amino acid N-terminal (red) and C-terminal regions (blue) bordering the homeodomain of anterior (M1-3) Hox, central Hox (M4-7), posterior Hox (M8-9) Cdx (M10-11), Xlox (M12), Evx (M13-14), Mox/Meox (M15), and Ro (M16). Color key for taxon groups at top of the figure. 20 aa terminal sequences and annotated HOX-L motifs available in the Supplementary material.

The ancestral Hox of the Last Common Xenacoelomorpha Ancestor (LC-XA) was proposed to consist of a single anterior, central and posterior Hox gene (Pascual-Anaya et al. 2013). Our data shows strong support for a single antHox and postHox gene in the LC-XA (Fig. 3). The ancestral antHox was possibly accompanied by the N-terminal KRSQP and the C-terminal KEGK motifs (Ueki et al. 2019; Singh et al. 2020), as both were identified across the three xenacoelomorph branches and at least one nephrozoan. The ancestral postHox was likely accompanied by the N-terminal WL/WM motif. However, the placement of the three *Xenoturbella* centHox genes suggests that at least three central genes (Fig. 3), accompanied by the ubiquitous pan-bilaterian N-terminal WM motif and one containing the shared acoel-deuterostome C-terminal NLKSMSQ motif, were present in the LC-XA. Regarding the ParaHox genes, the LC-XA likely possessed single copies of the Cdx, accompanied by the N-terminal TGKTRT motif, and Gsx genes, with the loss of an Xlox/Pdx homolog predating the diversification of xenacoelomorphs. Similarly, our results support a LC-XA containing single copies of all the extended Hox genes: Evx accompanied by the C-terminal WPYLDPYYAYLVSR motif, Meox, Mnx, Gbx, and Ro, the latter flanked by the C-terminal KAQLDQQ motif.

### Implications of Hox Data on Placement of Xenacoelomorpha

It remains an open question how contemporary Hox data can contribute to resolving Xenacoelomorpha's internal phylogeny or its position within Bilateria. This is often contingent on the dynamic position of *Xenoturbella*, which has been suggested to occupy several different positions: (i) with Acoelomorpha forming the sister group of Nephrozoa (Hejnol et al. 2009; Nakano et al. 2013; Cannon et al. 2016; Laumer et al. 2019), (ii) as a separate branch within the deuterostomes (Perseke et al. 2007), (iii) as a sister group to Ambulacraria separate to Acoelomorpha (Bourlat et al. 2003; Bourlat et al. 2006; Dunn et al. 2008; Bourlat et al. 2009), (iv) within Xenacoelomorpha as a sister group to Ambulacraria inside monophyletic Deuterostomia (Philippe et al. 2011; Philippe et al. 2019), or (v) inside Xenacoelomorpha as sister to Ambulacraria with Deuterostomia paraphyletic or even polyphyletic (Kapli et al. 2021).

Recently, Redmond (2024) presented an alternate hypothesis where Acoela was recovered with *Xenoturbella* in a new clade “Xenacoela,” as sister group to Nemertodermatida, challenging the current concept of Acoelomorpha (Hejnol et al. 2009; Mwinyi et al. 2010; Cannon et al. 2016; Laumer et al. 2019; Álvarez-Presas et al. 2024). We did not identify motif content as informative for resolving neither the relationships for Xenacoelomorpha nor its position in the animal tree. Conservation of motif content between Acoela and *Xenoturbella* to the exclusion of Nemertodermatida would support the “Xenacoela” scenario (Redmond 2024). However, we have not observed any derived motifs that support this hypothesis. On the contrary, the expanded set of central Hox genes remains unique to *Xenoturbella*, and the unique centHox2 KIGNKRSVDYAESGSTSPPS motif is absent from either clade of Acoelomorpha in conflict with the Xenacoela hypothesis.

**Fig. 4.**
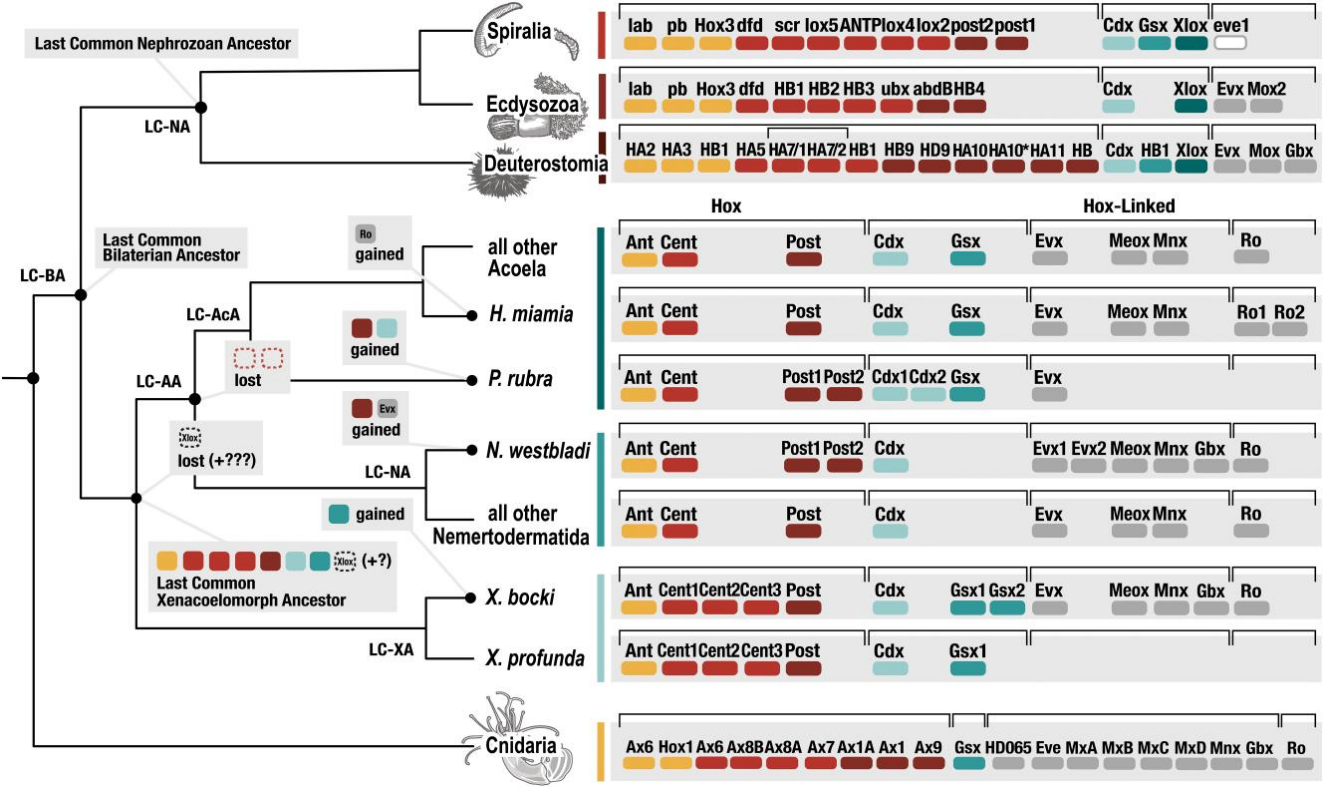
Description of Hox complement evolution in Xenacoelomorpha. Hypothesis on the gains and losses of HOX-L genes from the Last Common Xenacoelomorpha Ancestor (LC-XA) and Last Common Acoelomorph Ancestor (LC-AA). Colored markers denote different genes present in lineage: yellow (anterior Hox), red (central Hox), dark red (posterior Hox), light blue (Cdx), blue (Gsx), dark blue (Xlox), and gray (the extended genes Evx, Meox, Mnx, Gbx, and Ro). Marker with black dotted outline represents the absent Xlox/Pdx gene not observed in Xenacoelomorpha.

Additionally, all the motifs we described in Xenacoelomorpha are either lineage-specific or shared between all three groups. Under the “Xenambulacraria” hypothesis (Philippe et al. 2011), more Hox content shared between Xenacoelomorpha and Deuterostomia would be expected than with other groups. Although the presence of the acoel centHox NLKSMSQ motif in deuterostome HoxA5/Hox5 genes could be considered to fulfill this criterion, its significance is diluted when considering that the antHox KEGKL and the Cdx RK motifs (Fig. 4) could conversely support the hypothesis that xenacoelomorphs are sister group to the protostomes (Rouse et al. 2016). We observed no motifs shared uniquely between Xenacoelomorpha and Ambulacraria to the exclusion of chordates. The high degree of Hox content conserved within Xenacoelomorpha, combined with the absence of a convincing overlap with nephrozoan Hox content, therefore remains suggestive of a monophyletic Xenacoelomorpha outside of Nephrozoa (Cannon et al. 2016; Álvarez-Presas et al. 2024).

## Conclusion

The conservation of HOX-L (Hox, ParaHox, and extended Hox) content makes them one of the most intriguing leads for studying evolution of early Bilateria, particularly across the phylogenetic diversity of Xenacoelomorpha (Thomas-Chollier and Martinez 2016). The broad taxon sampling used here reveals surprising clade-specific Hox variation in this group and provides new insight on the possible ancestral state and evolution of the xenacoelomorph Hox complement. The presence of duplicate Hox genes in Acoelomorpha and *Xenoturbella* raises several questions about their origins—whether from lineage-specific gene duplications (*N. westbladi*), possible whole-genome duplications (Acoela), or the retention of ancestral content lost in other lineages (*Xenoturbella*). Despite the challenges of assessing Hox relationships between xenacoelomorphs and nephrozoans, our results support the homology of the xenacoelomorph antHox gene with nephrozoan Hox1, while the relationships of centHox and postHox remain ambiguous due to weak phylogenetic support and obscured by time.

We identified a particularly high proportion of shared Hox motif content between Xenacoelomorpha and Nephrozoa, including several motifs previously annotated as specific to Xenacoelomorpha. This suggests a deeper retention of shared Hox homeodomain content from the ancestral bilaterian than previously considered. In conjunction with our findings, the remaining disparity between xenacoelomorph and nephrozoan Hox genes suggests a scenario of both significant gene divergence and loss following the radiation of Bilateria. Given the challenge in capturing historical signal in Hox that have since been unrecognizably changed or lost, these scenarios should be revisited with additional genomic data before reconstructing ancestral bilaterian content. To the ongoing question of Xenacoelomorpha's phylogenetic position, neither our phylogenetic nor our motif analyses of contemporary xenacoelomorph Hox provide support for either “Xenacoela” nor the “Xenambulacraria” hypotheses.

## Materials and Methods

### Sampling and Dataset Compiling

We compiled our dataset from 40 xenacoelomorph transcriptome assemblies (Abalde and Jondelius 2025) and four genomes from *H. miamia* (Gehrke et al. 2019), *N. westbladi* (Abalde et al. 2023), *P. naikaiensis* (Arimoto et al. 2019), and *X. bocki* (Schiffer et al. 2024), while the reference dataset (Supplementary material) is built on a matrix of already annotated metazoan Hox and ParaHox homeodomains available on NCBI GenBank (Clark et al. 2016). This includes the already annotated Hox and ParaHox genes from the xenacoelomorphs *C. longifissura* (Hejnol and Martindale 2009), *S. roscoffensis* (Moreno et al. 2009), and *X. bocki* (Brauchle et al. 2018) and Hox data from a chordate (*Branchiostoma floridae*, with one *B. lanceolatum* sequence supplementing Hox5), a mollusk (*L. gigantea*, with one *L. goshimai* sequence supplementing Hox3), an annelid (*Capitella teleta*), a nematode (*Caenorhabditis elegans*), an arthropod (*D. melanogaster*, with one *Tribolium castaneum* sequence supplementing the arthropod zen/Hox3), one echinoderm (*Strongylocentrotus purpuratus*), one priapulid (*Priapulus caudatus*) and one cnidarian (*Nematostella vectensis*). We included rough genes from the mollusk *Haliotis asinina* and the echinoderm *Holothuria leucospilota* in the absence of available annotations from *L. gigantea* and *S. purpuratus*. We replaced the highly derived *D. melanogaster* zen-genes zen1 and zen2 with those from *T. castaneum* as was done previously (Ryan et al. 2007).

### Hox, ParaHox, and Extended Hox Annotations

We annotated xenacoelomorph homeodomains using the canonical Hox classifications found in Holland (2013): antHox (Hox1-3), centHox (Hox4-8), and postHox for Hox9-13(15), along with standard naming for the ParaHox (Cdx, Gsx, Xlox) and extended Hox genes (Evx, Meox/Mox, Mnx, Gbx, and Ro). For nephrozoan species with distinct Hox nomenclature (e.g. zen, mab, etc.), we included both canonical and lineage-specific names in phylogenetic trees and in supplemental tables (Supplementary material). It should be noted that the naming convention for *N. vectensis* Hox (Anthox) represents “anthozoan Hox” and not “anterior Hox.”

We extracted open-reading frames (ORFs) from transcriptome assemblies using the TransDecoder package and predicted Hox, ParaHox, and extended Hox homeodomain content with the PFAM (Finn et al. 2016) and UniProt (Wu et al. 2006) databases. We then searched our candidate Hox homeodomain peptides against hd60.hmm, a 60-position Hox/ParaHox-associated hidden Markov model (HMM) profile (Zwarycz et al. 2016) constructed from the homeobox database HomeoDB (Zhong et al. 2008; Zhong and Holland 2011). We aligned our target domains and additional bilaterian references from GenBank to the HMM profile using hmm2aln.pl v1.01 (https://github.com/josephryan/hmm2aln.pl), a program which leverages the hmmsearch tool (Potter et al. 2018). We removed sequences with more than five gaps to exclude highly ambiguous sequences using nogaps.py (available in GitHub repository). We made an additional hmm2aln.pl search with hd60.hmm of translated versions of the *H. miamia*, *N. westbladi* and *X. bocki* genome assemblies. We removed all non-HOX-L genes by constructing a maximum likelihood (ML) tree with a reference dataset of cnidarian Hox genes (Steinworth et al. 2023) and pruning all sequences that lay outside the smallest possible clade including *N. vectensis* Hox, ParaHox, and extended Hox genes with make_subalignment v0.06 (https://github.com/josephryan/make_subalignment). We trimmed notable introns/insertions present in a few homeodomain sequences prior to our phylogenetic analysis—both the original and trimmed sequences are available in the Supplementary material.

### Phylogenetic Analysis

We inferred a ML phylogeny for Hox, ParaHox, and other associated Hox genes using an alignment of the annotated 60 amino acid homeodomains in IQTree v1.6.10 (Trifinopoulos et al. 2016; Minh et al. 2020). ModelFinder (Kalyaanamoorthy et al. 2017) determined LG + G4 to be the best amino acid substitution model. Nodal support was calculated from 10,000 ultrafast bootstrap replicates (Minh et al. 2013; Hoang et al. 2018). The final dataset included 19 acoel, five nemertodermatid, two xenoturbellid, four deuterostome, four ecdysozoan, four lophotrochozoan, and one anthozoan species and was rooted on an outgroup of Ro genes from xenacoelomorph, nephrozoan, and anthozoan annotations.

### Homeodomain Motif Analysis

To supplement the phylogenetic analyses, we conducted additional searches outside of the homeodomain for more information on Hox gene orthology. We dissected the flanking 20 amino acid C-terminal and N-terminal regions from the 60 amino acid homeodomains using a custom script get_upstream_hd_downstream.pl (available in GitHub repository). Sequences with N- and C-terminals not included in the motif analyses are noted on the tree. We then used the MEME motif recognition algorithm (Bailey et al. 2015) for detecting ungapped motifs in the flanking regions with the ZOOPS (zero or one occurrence per sequence) motif discovery sequence model (Bailey and Elkan 1994) and default parameters, returning the top ten possible motifs for the C- and N-terminus of each gene. We then aimed for sequences with a motif E-value threshold of 5.0E-02 and discarded all motifs that did not fulfill those parameters. Beyond heavy conservation, the variation in E-values can be attributed to short sequence length and amino acid variation surrounding the motif boundaries. As MEME's motif search parameters require more than eight amino acids, some N-terminal sequences with short flanking regions were supplemented with randomized amino acids using the custom script pad_w_random_amino_acids.pl (available in GitHub repository). These randomized characters were later removed for the final motif alignment files and figures.

## Supplementary Material

evag094_Supplementary_Data

## Data Availability

Hox, ParaHox, and associated Hox gene, motif content, and other supplemental materials are available at https://github.com/yannbuck/xenaHox/. Release v1.0 represents the state of the repository at publication and is available on Zenodo (doi: 10.5281/zenodo.19206082).
