# Supplementary figures and images for: Patterns of Conservation and Loss of Hox Genes in Xenacoelomorph Lineage Since Divergence From Last Common Bilaterian Ancestor
Source: Genome Biol Evol. 2026 Apr 10;18(4):evag094. doi: 10.1093/gbe/evag094 (PMC13125763; doi:10.1093/gbe/evag094)

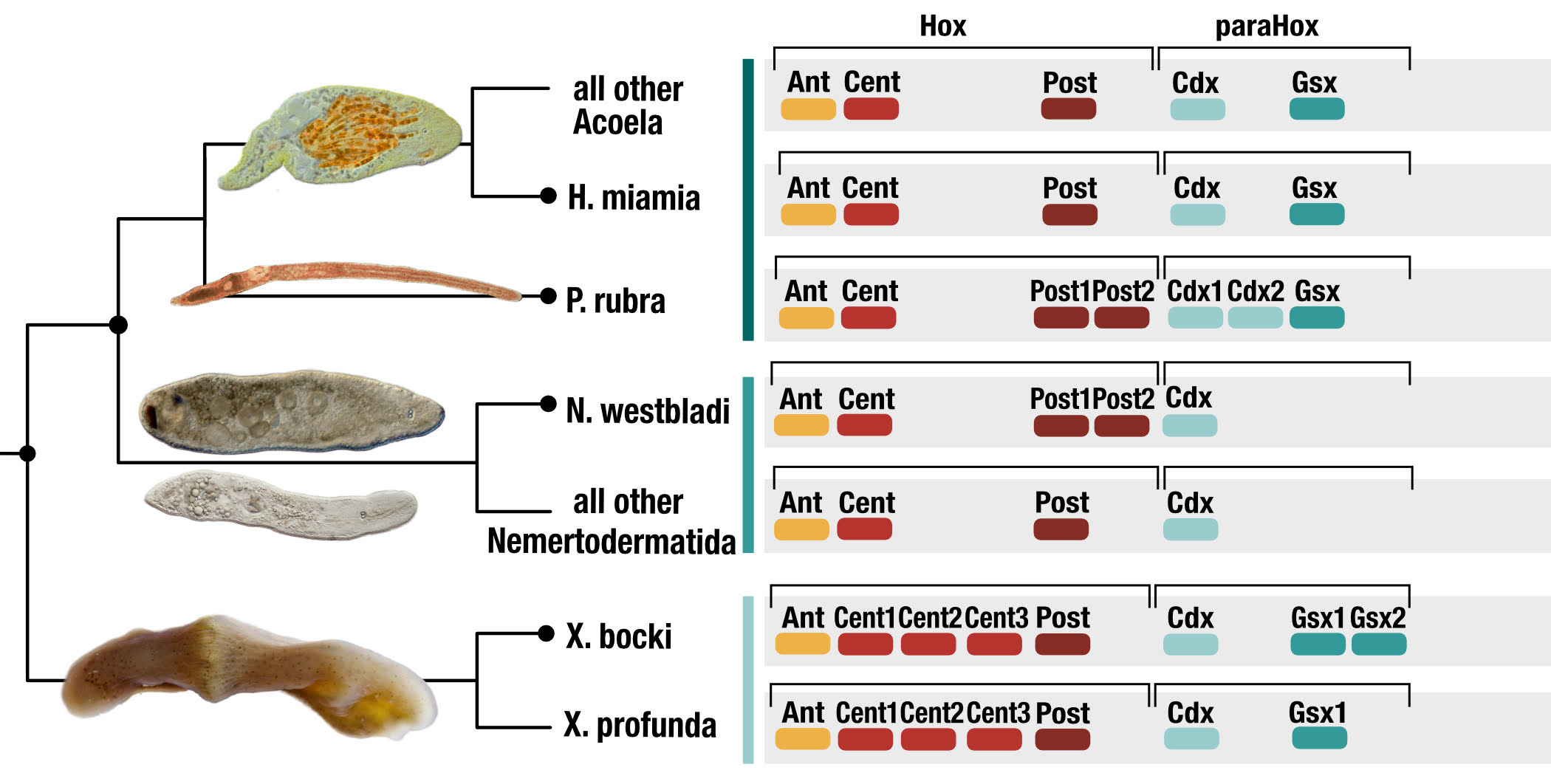

Supplement: evag094_Supplementary_Data [file evag094_supplementary_data.zip › xenaHox_promotional_image.jpg]
